# Traditional Systemic Treatment Options in Advanced Low-Grade Serous Ovarian Cancer after Successful Cytoreduction: A Systematic Review and Meta-Analysis

**DOI:** 10.3390/cancers14153681

**Published:** 2022-07-28

**Authors:** Rosa Montero-Macías, Pascal Rigolet, Elie Mikhael, Jonathan Krell, Vincent Villefranque, Fabrice Lecuru, Christina Fotopoulou

**Affiliations:** 1 Department of Obstetrics and gynecology, Centre hospitalier Simone-Veil, 95602 Eaubonne, France; elie.mikhael@ch-simoneveil.fr (E.M.); vincent.villefranque@ch-simoneveil.fr (V.V.); 2 Curie Institute, Paris-Saclay University, CNRS UMR 9187, Inserm U1196, CEDEX F-91898, 91400 Orsay, France; pascal.rigolet@u-psud.fr; 3 Department of Medical Oncology, Faculty of Medicine, Imperial College London, SW7 2AZ London, UK; j.krell@imperial.ac.uk; 4 Breast, Gynecology and Reconstructive Surgery Unit, Curie Institute, 75005 Paris, France; fabrice.lecuru@curie.fr; 5 Faculty of Medicine, Paris University, 75006 Paris, France; 6 Department of Gynaecologic Oncology, Faculty of Medicine, Imperial College London, SW7 2AZ London, UK; c.fotopoulou@imperial.ac.uk

**Keywords:** low-grade serous ovarian cancer, adjuvant treatment, chemotherapy, hormonal therapy

## Abstract

**Simple Summary:**

Low-Grade Serous Ovarian cancer (LGSOC) is considered less sensitive to traditional chemotherapy than its high-grade counterpart. Guidelines are still inconsistent around the use and value of cytotoxic and antihormonal agents in the adjuvant setting. Traditional cytotoxic or antihormonal systemic treatment option is not associated with a significant OS or PFS benefit in this Meta-analysis.

**Abstract:**

Objective: We performed a systematic literature review and a subsequent meta-analysis to compare traditional treatment options, i.e., antihormonal and cytotoxic, in LGSOC. Methods: We conducted a systematic literature review in MEDBASE and MEDLINE between September 2000 and June 2021 for women who received cytotoxic chemotherapy and/or antihormonal treatment after primary cytoreduction due to stage II–IV LGSOC and also at relapse. PFS and OS were calculated depending on the type of their adjuvant treatment. For each endpoint in the meta-analysis, pooled HR was calculated using the random effect model with the inverse variance weighted method. Only primary patients were included in the subsequent meta-analysis due to the small number of studies in the relapsed setting. Results: Five eligible first-line studies were included. Systemic chemotherapy failed to provide a significant OS benefit when compared to no systemic treatment (pooled HR = 1.01, 95% CI [0.79, 1.29]) after successful cytoreduction. Moreover, systemic chemotherapy followed by antihormonal treatment also did not result to a significant PFS or OS benefit when compared to systemic chemotherapy alone (for PSF: pooled HR = 0.59, 95% CI [0.33, 1.04]; for OS: pooled HR = 0.83, 95% CI [0.50, 1.39]). There were insufficient data from studies in the recurrent setting to allow their inclusion in the meta-analysis. Conclusions: In this meta-analysis, we failed to identify a traditional cytotoxic or antihormonal systemic treatment option that was associated with a significant OS or PFS benefit when administered following successful cytoreduction for advanced LGSOC. Prospective randomized studies are urgently warranted to define optimal adjuvant options in this challenging disease.

## 1. Introduction

Low-Grade Serous Ovarian Cancer (LGSOC) is a rare histological subtype of epithelial ovarian cancer. Approximately 90% of serous carcinomas are high-grade, and only 10% will be low-grade. For example, there are only around 560 cases of low-grade serous carcinomas diagnosed in the UK each year. Ref. [1] LGSOC is a distinct pathological and clinical entity and appears to exist on a continuum with their likely precursor lesions, the serous borderline tumors. It is characterized by a more indolent biological behavior and lower sensitivity to traditional chemotherapy (response rates of approximately 5%) when compared to high-grade serous ovarian cancer (HGSOC) [2].

Maximal effort cytoreductive surgery aimed at reduction of tumor load is the cornerstone of treatment for women with LGSOC. Attributed to its decreased chemosensitivity, debulking even to a residual disease less than 1 cm has been demonstrated to also be beneficial in those patients where total macroscopic clearance cannot be obtained [3]. For that reason, the value of neoadjuvant chemotherapy in LGSOC is not as well defined as in HGSOC [4].

Despite its indolent behavior, 70% of women with advanced stage LGSOC will experience a disease recurrence [5,6]. For that reason, NCCN [7] and ESMO [8] guidelines endorse therapeutic options that include systemic carboplatin and paclitaxel, either alone or with endocrine agents as maintenance in stage II–IV disease. Endocrine therapy alone, most commonly with an aromatase inhibitor, has also been considered. Currently, national guidelines and standards of practice vary broadly and personal preferences and views of the individual treating teams, often shape patients’ journeys without adequate evidence. Novel agents, such as MEK inhibitors, have shown significant promise, but funding and availability/licensing issues provide a significant obstacle to their wide incorporation into routine clinical care [2].

In the present work, we performed a literature review and, subsequently, a meta-analysis to evaluate the efficacy of traditional (i.e., cytotoxic and antihormonal) postoperative treatment options in patients with LGSOC.

## 2. Materials and Methods

### 2.1. Data Sources and Study Selection

A comprehensive, systematic computer literature search on PubMed was performed using the following Medical Subject Headings (MeSH) terms: “low grade serous ovarian cancer” AND/OR “adjuvant treatment” AND/OR “recurrent” AND/OR “chemotherapy” AND/OR “hormonal therapy”.

Separate searches were performed with MeSH terms on MEDLINE and EMBASE to extract all relevant literature available.

### 2.2. Study Selection

Inclusion criteria were studies published in English between September 2000 and June 2021. All types of meta-analyses, prospective, retrospective, and systematic reviews were eligible for inclusion in the current review and all retrieved studies were evaluated for eligibility by two authors (R.M. and E.M.)

The population of this study includes patients with histologically confirmed stage II–IV LGSOC who have undergone primary cytoreduction. We included studies that reported data about traditional adjuvant treatment approaches: cytotoxic chemotherapy, antihormonal agents, or a combination of both. Options of antihormonal agents included: aromatase inhibitors, tamoxifen, and LH-HR analogues.

### 2.3. We Excluded Studies That

- Did not report specific information about type of adjuvant treatment;
- Did not include survival data;
- Included patients who did receive neoadjuvant treatment;
- Included other new targeted therapies: Poly-ADP ribose polymerase (PARP) inhibitors, MEK inhibitors, and antiangiogenics;
- Included patients who received intraperitoneal chemotherapy;
- Grey Literature was not considered—case reports, letters, and comments were excluded.

Our Systematic review was submitted to the PROSPERO register and published in the database with the registration number: CRD42021277026.

### 2.4. Statistical Analysis

Age and follow-up time were expressed in mean or median depending on the study.

Survival was expressed as median time to recurrence, % patients, PFS or OS in 2, 3 or 5 years and HR for PFS and/or OS. This heterogeneity to describe survival made the data difficult to analyze.

Descriptive statistics were used to describe the treatments employed for the qualitative analysis, with median PFS and OS measurements reported individually along with their respective 95% CI.

After study selection and application of the exclusion criteria, we stratified studies included in the meta-analysis to systemic chemotherapy versus no treatment and to hormonal treatment after systemic chemotherapy versus systemic chemotherapy only. We could not identify any relevant literature evaluating antihormonal treatment alone versus no systemic treatment, nor any literature about chemotherapy versus antihormonal treatment. We used the R software version 3.6 with the metafor and the meta packages. For all the studies, we reported hazard ratios (HR) and we pooled the HR for OS and PFS using the inverse variance method (the weight given to each study being the inverse of the variance) and the random effects model to account for heterogeneity. We used the Sidik–Johnkman estimator for tau and Chi-squared and Cochrane Q-tests to quantify heterogeneity across studies by computing the I^2^ for each endpoint. All 95% CIs were two-sided.

### 2.5. Quality Assessment of Studies

The methodological quality of cohort studies was evaluated using the “Quality Assessment Tools of the National Heart, Lung, and Blood Institute” (NHLBI) [9].

## 3. Results

### 3.1. Evidence Acquisition

**Figure 1:**
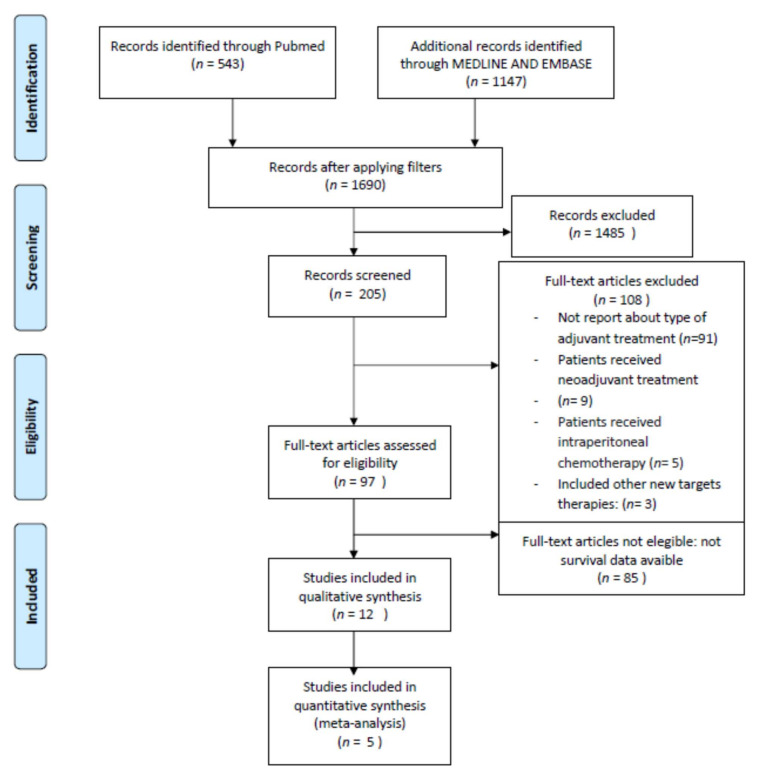
PRISMA Flow Diagram.

Searching in the Literature with MeSH terms we found 1690 articles. After the exclusion of Grey Literature, i.e., case reports, letters, and comments, and non-English paper, 205 records were selected. After reading the abstracts, 108 were excluded as per the aforementioned exclusion criteria. We assessed 97 full articles for eligibility and identified only 12 studies eligible for inclusion in the qualitative analysis (Figure 1). The other 85 articles did not provide survival data of the therapeutic strategy target of our study.

### 3.2. Study Characteristics

In the 12 studies eligible for inclusion, 9 concerned patients in the first line setting (Primary treatment) and 3 concerned patients with recurrent disease.

Except one phase II trial [10] all other studies were of retrospective nature. Two of the studies had a prospective part in addition to the retrospective analysis [11,12].

The baseline characteristics of the 12 included studies are shown in Table 1 (Primary treatment) and Table 2 (Recurrence).

Overall, 1892 patients were included from all studies: 1734 in the first-line setting and 158 in recurrent disease.

### 3.3. Qualitative Synthesis 

#### First-Line Setting (Primary Treatment)

Nine studies were included in this group. The type of treatment was antihormonal therapy alone in one study [19] and a combination of chemotherapy and antihormonal therapy in three studies [11,12,15], whereas two studies compared chemotherapy alone vs just watch and wait [13,14].

Three studies reported descriptive analysis data of patients treated with only chemotherapy [16,17,18].

In four these nine studies, only a descriptive analysis of treatments was done (three for cytotoxic chemotherapy, one for antihormonal treatment) without any control group and did not report any survival results for adjuvant treatment with HR [16,17,18,19]. As they also did not consistently report survival, these studies were not included in the meta-analysis.

The five remaining studies that were ultimately included in the present meta-analysis evaluated the following regimens: three studies compared cytotoxic chemotherapy alone vs chemotherapy followed by maintenance antihormonal therapy [11,12,15] and two studies compared chemotherapy vs no adjuvant treatment at all [13,14]. We report in more detail the three studies that evaluated cytotoxic chemotherapy alone vs chemotherapy followed by maintenance antihormonal therapy below.

Schlumbrecht et al. (2011) divided antihormonal therapy into two categories. The first category, named “Consolidation therapy”, included patients without radiological or clinical evidence of residual disease at the end of their first-line chemotherapy, who then received antihormonal treatment. The second category, named “Maintenance therapy”, included patients who had persistent but stable disease after adjuvant chemotherapy, who then received antihormonal treatment. We opted to include only the data of patients in the category “Consolidation therapy”, to be consistent with the rest of the included patients in this meta-analysis that had undergone complete cytoreduction. In total, 95.8% of the patients were treated with platinum-based chemotherapy [15].

Gershenson et al. (2017) evaluated patients who underwent primary cytoreductive surgery followed by platinum-based chemotherapy and observation versus antihormonal therapy (as per the physician’s choice) starting within 3 months of completion of postoperative chemotherapy. In total, 54.3% of the patients received Letrozole [11].

A previous study conducted by Gershenson et al. in 2015 included a subgroup of 287 patients who had undergone primary cytoreductive surgery followed by platinum-based chemotherapy. In total, 50 of these patients also received maintenance hormonal therapy, which we also included in the present analysis [12].

The studies and their regimens are presented in Table 3.

### 3.4. Recurrent Setting

Only three case studies were identified and included in this group: 36 patients reported by Tang et al. (2019) [10], 64 patients by Gershenson et al. (2012) [20], and 58 by Gershenson et al. (2009) [6].

The types of applied treatments were antihormonal treatment alone in two studies (Tang et al., 2019, Gershenson et al., 2012) [10,20] and chemotherapy alone in one study (Gershenson 2009) [6]. These three studies were only descriptive and did not provide any comparative data with a control group. None of these studies included HR in their results.

In the PARAGON phase II study (Tang et al., 2019) anastrozole given for at least 6 months was associated with a clinical benefit rate of 61% in patients with recurrent ER- and/or PR-positive low-grade ovarian cancer or serous ovarian borderline tumors. The toxicity profile reported was acceptable toxicity. Median PFS was 11.1 months (95% CI: 3.2–11.9) [10].

In a further retrospective study by Gershenson et al. (2012) evaluating 64 patients with histologically confirmed, recurrent low-grade serous ovarian/peritoneal carcinoma who received hormonal therapy at their institution between 1989 and 2009 showed an overall response rate of only 9%. In total, 61% of the patient regimens resulted in a progression-free survival duration of at least 6 months. Patient regimens involving ER+/PR+ disease produced a longer median time to disease progression (8.9 months) than patient regimens involving ER+/PR- disease did (median = 6.2 months; *p* = 0.053). This antitumor activity was considered as moderate by the authors and appealed for further studies to determine whether ER/PR expression status may be used as a predictive biomarker for low-grade disease [20].

In 2009, Gershenson et al. performed a descriptive analysis of 58 patients with recurrent LGSOC, who received 108 combinations of different chemotherapy regimens (“patient regimens”); 60.6% were platinum-based. Stable disease was observed in 65 patients (60.2%). The overall response rate for the platinum-sensitive cohort was 4.9%, and 2.1% for the platinum-resistant cohort. The median overall survival was 87.1 months and the median time to progression was 29.0 weeks (34.7 weeks for platinum-sensitive cohort and 26.4 weeks for platinum-resistant cohort) (*p* = 0.32) [6].

With only three studies in this group, a valid meta-analysis was not feasible. Thus, we only included first-line treatment data in this meta-analysis.

### 3.5. Quantitative Synthesis

For the primary patients who were included in the present meta-analysis, median OS for those treated with systemic chemotherapy alone ranged between 88.2 and 106.8 months, whereas for those patients who were additionally treated with antihormonal treatment as a maintenance, the median OS ranged between 100.9 and 115.7 months and the median PFS between 25.9 and 76.4 months. Median PFS was 25.9 and 76.4 months, respectively.

**Figure 2:**
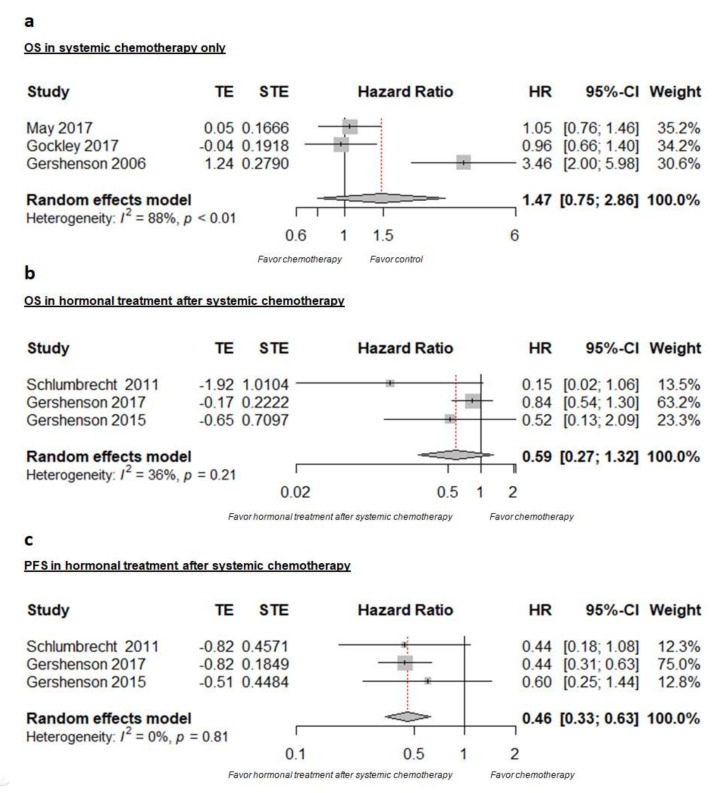
Meta-analysis for: (**a**) OS in patients with systemic chemotherapy alone, (**b**) for OS and (**c**) PFS for patients treated with additional antihormonal maintenance treatment after completion of systemic chemotherapy.

Forest plots summarizing the efficacy of the various treatment approaches are presented in Figure 2. The pooled HR analyzes did not reveal any statistically significant PFS or OS benefit associated with the addition of antihormonal treatment after completion of systemic first-line chemotherapy in LGSOC patients who had undergone complete cytoreduction when compared to systemic chemotherapy alone: for PFS, pooled HR = 0.59, 95% CI [0.33, 1.04]), I^2^ = 77% (Figure 2c), and for OS, pooled HR = 0.83, 95% CI [0.50, 1.39], I^2^ = 46% (Figure 2b).

Moreover, systemic chemotherapy also failed to significantly improve OS when compared to no treatment at all after successful cytoreduction (pooled HR = 1.01, 95% CI [0.79, 1.29], I^2^ = 0%, Figure 2a). No PFS data were reported for the studies with chemotherapy alone.

### 3.6. Quality Assessments

The quality of 11 retrospective studies and of the prospective study were assessed using the NHLBI study quality assessment tool and were rated as “fair” in all cases. The most common biases were the absence of sample size justification and the missed measurement of confounding variables. Table 4

## 4. Discussion

To our knowledge, this is the first comprehensive systematic review and meta-analysis to assess the potential survival impact of antihormonal maintenance treatment or even cytotoxic first-line chemotherapy in patients who had undergone successful cytoreduction for advanced stage LGSOC. Analyzed evidence failed to demonstrate a survival benefit through the addition of antihormonal agents after completion of first-line chemotherapy compared to chemotherapy alone in completely cytoreduced LGSOC patients. Equally, chemotherapy alone was not associated with any significant improvement in survival compared to watch and wait strategies.

These data give a clear signal of the necessity of overcoming conventional ways of thinking for the management of LGSOC. It is prime time for traditional cytotoxic and antihormonal agents to give their place to novel targeted approaches, especially developed for these rare neoplasms in an overall scheme of individualization of treatment.

Chemosensitivity in LGSOC is being increasingly disputed [17,21]. Conventional chemotherapy studies for advanced ovarian cancer used to include all histological subtypes, i.e., both low and high grade, without specific differentiation. However, in accordance with the universal trends in oncology to individualize care, systemic studies also in ovarian cancer have started stratifying patients according to their tumor biology and even the development of umbrella studies aimed at recruiting across various organs by equivalent histology.

In surgical studies, such a differentiation is more challenging due to the well-defined difficulties of objectively performed high quality surgical studies. For example, the prospective phase III randomized GOG 0213 trial included only seven patients with LGSC [22]. Similarly, the European study DESKTOP III also recruited only 10 LGSC patients [23].

Antihormonal treatment in ovarian cancer has been part of the treatment armamentarium for years, even for high-grade histology. For example, Tamoxifen has been broadly accepted as an alternative to non-platinum monotherapy for palliative relapsed patients [24]. Patients with LGSOC with higher estrogen receptor (ER) expression appear to benefit from such an approach even in earlier treatment settings [25]. Still, solid data from prospective randomized clinical trials of antihormonal treatment versus cytotoxic chemotherapy are lacking and broad national and international variations in practice exist regarding their clinical utilization. For example, in a German survey, 43% of physicians (gynecologists, gynecologic oncologists, and oncologists) did not consider antihormonal therapy as a treatment option [26].

Studies of the molecular biology of LGSOC have identified a high frequency of estrogen and progesterone expression, but also the important role of the mitogen-activated protein kinase (MAPK) signaling pathway on its pathogenesis. *KRAS* mutations occur in 16% to 44% of LGSOCs, *BRAF* mutations in 2% to 20%, and *NRAS* mutations in up to 26%. This differentiates LGSOC from HGSOC, the latter being associated with ubiquitous p53 mutations, copy number abnormalities, and DNA repair defects [27].

MEK inhibitors are orally bioavailable, small-molecule inhibitors of MEK1/2. An initial phase II trial examined selumetinib in recurrent LGSOC and demonstrated objective response rates of 15% and a median PFS of 11.0 months. No correlation between response and mutational status was found [28].

Two large randomized clinical trials of MEK inhibitors in recurrent LGSOC followed this initial study. GOG 0281 was a phase II/III trial comparing trametinib with the physician’s choice of pegylated liposomal doxorubicin or weekly paclitaxel, topotecan, letrozole, or tamoxifen [29]. MILO/ENGOT-ov11 compared binimetinib with the physician’s choice [30].

On the basis of an interim analysis of 303 patients, enrolment in MILO was discontinued following an interim analysis as the PFS HR crossed the predefined futility boundary. In an updated analysis, median PFS times were 10.4 for binimetinib and 11.5 months for the physician’s choice (HR, 1.15; *p* = 0.748). However, GOG 0281 successfully met its primary end point, with a median PFS of 13.0 months for trametinib and 7.2 months for the physician’s choice (HR, 0.48; *p* < 0.001) and ORRs of 26% and 6.2% for trametinib and the physician’s choice, respectively. In England, trametinib is currently funded via the Cancer Drugs Fund for patients with recurrent LGSOC who have progressive disease following platinum-based chemotherapy and who have exhausted all endocrine therapy. Data to support the use of MEK inhibitors in the first-line setting are still lacking and further studies are required. Furthermore, studies of MEK inhibitors in combination with other targeted therapies such as BRAF inhibitors are showing promise in LGSOC [30,31].

The strength of our work is that it is the first meta-analysis in the literature about systematic adjuvant treatment in LGSOC, but there are several limitations to our study. The most important is that very few studies are available, and the heterogeneity among these studies limits a uniform interpretation of the results and the inclusion of the data in quantitative analyses. More data are now needed to confirm that hormonal treatment after systemic chemotherapy in LGSOC does not lead to any benefit for OS or for PSF and to determine the best way to combine these two therapies, if applicable.

In this context, the multicenter prospective randomized phase III trial NRG-GY-019, which started recruiting in mid-2019, randomizes newly diagnosed stage II–IV LGSOC-patients following initial debulking surgery to adjuvant platinum/taxane chemotherapy followed by maintenance letrozole vs letrozole alone [32]. However, more randomized trials are necessary to have scientific evidence about the best approach in this subgroup of patients.

## 5. Conclusions

Our data reinforce current evidence that future treatment trends are clearly pointing towards the omission of traditional systemic approaches in, at least optimally cytoreduced, LGSOC patients. This approach would spare unnecessary toxicity for many patients. Our focus should concentrate on the development of especially targeted agents and their broad implementation, even in restricted settings.

## Figures and Tables

**Table 1:** Summary of the baseline characteristics of the nine included studies for the first-line setting (primary treatment).

Study (Year)	Design	Setting	Arms	Follow-up (Months)	Patients (*n*)	Population	Age (Years)	Histology
May (2017) [13]	Retrospective	multicenter	adjuvant chemo vs no adjuvant chemo	Median 58.8	439	Stage I–IV with PCRS	Median 54	LGSOC
Gockley (2017) [14]	Retrospective	National Cancer Database	adjuvant chemo vs no adjuvant chemo	Median 72.9	280	Stage IIIc–IV with PCRS	Mean 53.6	LGSOC
Schlumbrecht (2011) [15]	Retrospective	Single Cancer Center (MDACC)	adjuvant chemo + hormonal therapy vs adjuvant chemo only	Median 60.9	194	Newly diagnosed PCRS	Mean 44.9	LGSOC
Gershenson (2017) [11]	Retrospective and prospective	Single Cancer Center (MDACC)	adjuvant chemo + hormonal therapy vs adjuvant chemo only	Median 70.8	203	Stage II–IV with PCRS	Median 47.5	LGSOC LGSPC
Gershenson (2015) [12]	Retrospective and prospective	Single Cancer Center (MDACC)	adjuvant chemo + hormonal therapy vs adjuvant chemo only	Median 72.5	287	Stage I–IV with PCRS	Median 46.1	LGSOC GSPC
Grabowski (2016) [16]	Retrospective	AGO Database of 4 RT	Adjuvant chemo	---	145	Stage IIIb–IV with CCRS	Median 48	LGSOC
Gershenson (2006) [17]	Retrospective	Single Cancer Center (MDACC)	Adjuvant chemo	Mean 71	112	Stage II–IV with PCRS	Median 43	LGSOC
Fader (2013) [18]	Retrospective	multicenter	Adjuvant chemo	Median 47.1	47	stage III–IV with MRCRS	Median 56.5	LGSOC
Fader (2017) [19]	Retrospective	multicenter	Adjuvant hormonal therapy	Median 41	27	Stage II–IV with PCRS	Median 47.5	LGSOC

HT = Hormonal therapy, HC = Hormonal consolidation, ACT: Adjuvant Chemotherapy, PBACT: Platinum-Based Adjuvant Chemotherapy, RT: Randomized Trial, CCRS: complete cytoreductive surgery, MRCRS: Microscopic residual cytoreductive surgery, PCRS: Primary Cytoreductive surgery, TTP: Time to Progression, LGSOC: Low-Grade Serous Ovarian Cancer, LGSPC: Low-Grade Serous Peritoneal Cancer.

**Table 2:** Summary of the baseline characteristics of the three included studies for recurrence treatment.

Study (Year)	Design	Setting	Arms	Follow-up (Months)	Patients (*n*)	Population	Age (Years)	Histology
**Tang** **(2019)** [10]	Phase II trial	Multicenter	Only HT (anastrozole)	Mean 31.1	36	Recurrent LGSOC	Mean 57	LGSOC
**Gershenson (2012)** [20]	Retrospective	Single Cancer Center	Only HT	-	64	Recurrent LGSOC and LGSPC	Median 49.4	LGSOC LGSPC
**Gershenson (2009)** [6]	Retrospective	Single Cancer Center	Only chemo	-	58	Recurrent LGSOC	Median 43.2	LGSOC

HT = Hormonal therapy, LGSOC: Low-Grade Serous Ovarian Cancer, LGSPC: Low-Grade Serous Peritoneal Cancer.

**Table 3:** Summary of the qualitative characteristics of the 12 included studies for primary and recurrence treatment.

Study, Date	Control Arm	Intervention Arm	Primary Endpoint	Median PFS (Mo)	HR	95%CI	Median OS (Mo)	HR	95% CI
PRIMARY									
May 2017 [13]	no AC (269)	PBAC (170)	Response to ACT	-	-	-	106.8	1.05	0.76–1.46
Gockley 2017 [14]	no AC (140)	PBAC (140)	OutcomeSurvival factors	-	-	-	88.2	0.96	0.66–1.40
Schlumbrecht 2011 [15]	ACT (170)	ACT +HC (9)	effect of demographics and treatment on survival	76.4	0.44	0.18–1.08	-	0.15	0.02–1.06
Gershenson 2017 [11]	PBACT (133)	PBACT +HMT (70)	outcome	64.9	0.44	0.31–0.64	115.7	0.84	0.54–1.30
Gershenson 2015 [12]	ACT (159)	ACT +HMT (50)	outcome	-	0.92	0.64–1.32	-	1.05	0.64–1.73
Grabowski 2016 [16]	-	PBACT (145)	efficacy of PBACT after CCRS	92.0	-	-	97.0	-	-
Gershenson 2006 [17]	-	PBACT (112)	Clinical Behavior Analysis	19.5	-	-	81.8	-	-
Fader 2013 [18]	-	PBACT (47)	Evaluation of Clinicopathological variables	33.2			96.9		
Fader 2017 [19]	-	PCRS + HT (26)NACT + PCRS + HT (1)	Outcome	-	-	-	-	-	-
RECURRENT DISEASE									
Tang 2019 [10]	-	Anastrozole	Clinical Benefit Rates	11.1	-	3.2–11.9	-	-	-
Gershenson 2012 [20]	-	HT	Efficacy of HT	-	-	-	78.2	-	-
Gershenson 2009 [6]	-	Chemotherapy	Evaluate chemoresistance of recurrent LGSOC	-	-	-	87.1	-	56.8–117.3

HT = Hormonal therapy, HC = Hormonal consolidation, ACT: Adjuvant Chemotherapy, PBACT: Platinum-Based Adjuvant Chemotherapy, RT: Randomized Trial, CCRS: complete cytoreductive surgery, MRCRS: Microscopic residual cytoreductive surgery, PCRS: Primary Cytoreductive surgery, TTP: Time to Progression, LGSOC: Low-Grade Serous Ovarian Cancer, LGSPC: Low-Grade Serous Peritoneal Cancer.

**Table 4:** Quality assessment of the 12 included studies.

Study, Year	Criteria													
	1	2	3	4	5	6	7	8	9	10	11	12	13	14
Primary Treatment														
May, 2017 [13]							NA	NA		NA		NA	NA	
Gockley, 2017 [14]							NA	NA		NA		NA	NA	
Schlumbrecht, 2011 [15]							NA	NA		NA		NA	NA	
Gershenson, 2017 [11]							NA	NA		NA		NA	NA	
Gershenson, 2015 [12]							NA	NA		NA		NA	NA	
Grabowski, 2016 [16]							NA	NA		NA		NA	NA	
Gershenson, 2006 [17]							NA	NA		NA		NA	NA	
Fader, 2013 [18]							NA	NA		NA		NA	NA	
Fader, 2017 [19]							NA	NA		NA		NA	NA	
Recurrence Treatment														
Tang, 2019 [10]							NA	NA		NA		NA	NA	
Gershenson, 2012 [20]							NA	NA		NA		NA	NA	
Gershenson, 2009 [6]							NA	NA		NA		NA	NA	

1. Was the research question or objective in this paper clearly stated? 2. Was the study population clearly specified and defined? 3. Was the participation rate of eligible persons at least 50%? 4. Were all the subjects selected or recruited from the same or similar populations (including the same time period)? Were inclusion and exclusion criteria for being in the study prespecified and applied uniformly to all participants? 5. Were sample size justification, power description, or variance and effect estimates provided? 6. For the analyses in this paper, were the exposure(s) of interest measured prior to the outcome(s) being measured? 7. Was the timeframe sufficient so that one could reasonably expect to observe an association between exposure and outcome if it existed? 8. For exposures that can vary in amount or level, did the study examine different levels of the exposure as related to the outcome (e.g., categories of exposure, or exposure measured as continuous variable)? 9. Were the exposure measures (independent variables) clearly defined, valid, reliable, and implemented consistently across all study participants? 10. Was the exposure(s) assessed more than once over time? 11. Were the outcome measures (dependent variables) clearly defined, valid, reliable, and implemented consistently across all study participants? 12. Were the outcome assessors blinded to the exposure status of participants? 13. Was loss to follow-up after baseline 20% or less? 14. Were key potential confounding variables measured and adjusted statistically for their impact on the relationship between exposure(s) and outcome(s)? Legend: yes: 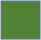, no: 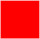.

## References

[1] Malpica A., Deavers M.T., Lu K., Bodurka D., Atkinson E.N., Gershenson D.M., Silva E.G. (2004). Grading Ovarian Serous Carcinoma Using a Two-Tier System. Am. J. Surg. Pathol..

[2] Gadducci A., Cosio S. (2020). Therapeutic Approach to Low-Grade Serous Ovarian Carcinoma: State of Art and Perspectives of Clinical Research. Cancers.

[3] Bogani G., Maggiore U.L.R., Paolini B., Diito A., Martinelli F., Lorusso D., Raspagliesi F. (2019). The detrimental effect of adopting interval debulking surgery in advanced stage low-grade serous ovarian cancer. J. Gynecol. Oncol..

[4] Cobb L.P., Sun C.C., Iyer R., Nick A.M., Fleming N.D., Westin S.N., Sood A.K., Wong K.K., Silva E.G., Gershenson D.M. (2020). The role of neoadjuvant chemotherapy in the management of low-grade serous carcinoma of the ovary and peritoneum: Further evidence of relative chemoresistance. Gynecol. Oncol..

[5] Crane E.K., Sun C.C., Ramirez P.T., Schmeler K.M., Malpica A., Gershenson D.M. (2014). The role of secondary cytoreduction in low-grade serous ovarian cancer or peritoneal cancer. Gynecol. Oncol..

[6] Gershenson D.M., Sun C.C., Bodurka D., Coleman R.L., Lu K.H., Sood A.K., Deavers M., Malpica A.L., Kavanagh J.J. (2009). Recurrent low-grade serous ovarian carcinoma is relatively chemoresistant. Gynecol. Oncol..

[7] Armstrong D.K., Alvarez R.D., Bakkum-Gamez J.N., Barroilhet L., Behbakht K., Berchuck A., Chen L.M., Cristea M., DeRosa M., Eisenhauer E.L. (2021). Ovarian Cancer, Version 2.2020, NCCN Clinical Practice Guidelines in Oncology. J. Natl. Compr. Canc. Netw..

[8] Colombo N., Sessa C., Du Bois A., Ledermann J., McCluggage W.G., McNeish I., Morice P., Pignata S., Ray-Coquard I., Vergote I. (2019). ESMO–ESGO consensus conference recommendations on ovarian cancer: Pathology and molecular biology, early and advanced stages, borderline tumours and recurrent disease. Ann. Oncol..

[9] Study Quality Assessment Tools|NHLBI, NIH. https://www.nhlbi.nih.gov/health-topics/study-quality-assessment-tools.

[10] Tang M., O’Connell R.L., Amant F., Beale P., McNally O., Sjoquist K.M., Grant P., Davis A., Sykes P., Mileshkin L. (2019). PARAGON: A Phase II study of anastrozole in patients with estrogen receptor-positive recurrent/metastatic low-grade ovarian cancers and serous borderline ovarian tumors. Gynecol. Oncol..

[11] Gershenson D.M., Bodurka D., Coleman R.L., Lu K.H., Malpica A., Sun C.C. (2017). Hormonal Maintenance Therapy for Women With Low-Grade Serous Cancer of the Ovary or Peritoneum. J. Clin. Oncol..

[12] Gershenson D.M., Bodurka D.C., Lu K.H., Nathan L.C., Milojevic L., Wong K.K., Malpica A., Sun C.C. (2015). Impact of Age and Primary Disease Site on Outcome in Women With Low-Grade Serous Carcinoma of the Ovary or Peritoneum: Results of a Large Single-Institution Registry of a Rare Tumor. J. Clin. Oncol..

[13] May T., Lheureux S., Bernardini M., Jiang H., Tone A. (2017). Clinical behavior of low grade serous ovarian carcinoma: Ananalysis of 714 patients from the Ovarian Cancer Association Consortium (OCAC). Gynecol. Oncol..

[14] Gockley A., Melamed A., Bregar A.J., Clemmer J.T., Birrer M., Schorge J.O., Del Carmen M.G., Rauh-Hain J.A. (2017). Outcomes of Women With High-Grade and Low-Grade Advanced-Stage Serous Epithelial Ovarian Cancer. Obstet. Gynecol..

[15] Schlumbrecht M.P., Sun C.C., Wong K.N., Broaddus R.R., Gershenson D.M., Bodurka D.C. (2011). Clinicodemographic factors influencing outcomes in patients with low-grade serous ovarian carcinoma. Cancer.

[16] Grabowski J.P., Harter P., Heitz F., Pujade-Lauraine E., Reuss A., Kristensen G., Ray-Coquard I., Heitz J., Traut A., Pfisterer J. (2016). Operability and chemotherapy responsiveness in advanced low-grade serous ovarian cancer. An analysis of the AGO Study Group metadatabase. Gynecol. Oncol..

[17] Gershenson D.M., Sun C.C., Lu K.H., Coleman R.L., Sood A.K., Malpica A., Deavers M.T., Silva E.G., Bodurka D.C. (2006). Clinical Behavior of Stage II-IV Low-Grade Serous Carcinoma of the Ovary. Obstet. Gynecol..

[18] Fader A.N., Java J., Ueda S., Bristow R.E., Armstrong D.K., Bookman M.A., Gershenson D.M., Gynecologic Oncology Group (GOG) (2013). Survival in Women With Grade 1 Serous Ovarian Carcinoma. Obstet. Gynecol..

[19] Fader A.N., Bergstrom J., Jernigan A., Tanner E.J., Roche K.L., Stone R.L., Levinson K.L., Ricci S., Wethingon S., Wang T.-L. (2017). Primary cytoreductive surgery and adjuvant hormonal monotherapy in women with advanced low-grade serous ovarian carcinoma: Reducing overtreatment without compromising survival?. Gynecol. Oncol..

[20] Gershenson D.M., Sun C.C., Iyer R.B., Malpica A.L., Kavanagh J.J., Bodurka D.C., Schmeler K., Deavers M. (2012). Hormonal therapy for recurrent low-grade serous carcinoma of the ovary or peritoneum. Gynecol. Oncol..

[21] Zantow E., Chen A., Zhao D., Mashburn S., Underwood H., Subia M.B., Zuna R., Moore K., Holman L. (2017). Clinical Factors Associated with Short- and Long-Term Survival in Low Grade Ovarian Carcinoma. Gynecol. Oncol..

[22] Coleman R.L., Spirtos N.M., Enserro D., Herzog T.J., Sabbatini P., Armstrong D.K., Kim J.-W., Park S.-Y., Kim B.-G., Nam J.-H. (2019). Secondary Surgical Cytoreduction for Recurrent Ovarian Cancer. N. Engl. J. Med..

[23] Harter P., Sehouli J., Vergote I., Ferron G., Reuss A., Meier W., Greggi S., Mosgaard B.J., Selle F., Guyon F. (2021). Randomized Trial of Cytoreductive Surgery for Relapsed Ovarian Cancer. N. Engl. J. Med..

[24] Tropé C., Marth C., Kaern J. (2000). Tamoxifen in the treatment of recurrent ovarian carcinoma. Eur. J. Cancer.

[25] Langdon S.P., Gourley C., Gabra H., Stanley B. (2016). Endocrine therapy in epithelial ovarian cancer. Expert Rev. Anticancer Ther..

[26] Alavi S., Harter P., Richter R., Keller M., Oskay Özcelik G., Mustea A., Schmalfeldt B., Wimberger P., Trillsch F., Mahner S. (2018). Monitor VII: Treatment Strategies of Low Grade Ovarian Carcinomas—A German Survey Of The Charité—Berlin and Kliniken Essen Mitte with Support of the Study Groups Noggo and Ago. Ann. Oncol..

[27] Hsu C.-Y., Bristow R., Cha M.S., Wang B.G., Ho C.-L., Kurman R.J., Wang T.-L., Shih I.-M. (2004). Characterization of Active Mitogen-Activated Protein Kinase in Ovarian Serous Carcinomas. Clin. Cancer Res..

[28] Farley J., Brady W., Vathipadiekal V., A Lankes H., Coleman R., Morgan M., Mannel R., Yamada S.D., Mutch D., Rodgers W.H. (2012). Selumetinib in women with recurrent low-grade serous carcinoma of the ovary or peritoneum: An open-label, single-arm, phase 2 study. Lancet Oncol..

[29] Gershenson D.M., Miller A., E Brady W., Paul J., Carty K., Rodgers W., Millan D., Coleman R.L., Moore K.N., Banerjee S. (2022). Trametinib versus standard of care in patients with recurrent low-grade serous ovarian cancer (GOG 281/LOGS): An international, randomised, open-label, multicentre, phase 2/3 trial. Lancet.

[30] Monk B.J., Grisham R.N., Banerjee S., Kalbacher E., Mirza M.R., Romero I., Vuylsteke P., Coleman R.L., Hilpert F., Oza A.M. (2020). MILO/ENGOT-ov11: Binimetinib Versus Physician’s Choice Chemotherapy in Recurrent or Persistent Low-Grade Serous Carcinomas of the Ovary, Fallopian Tube, or Primary Peritoneum. J. Clin. Oncol..

[31] Gershenson D.M., Gourley C., Paul J. (2020). MEK Inhibitors for the Treatment of Low-Grade Serous Ovarian Cancer: Expanding Therapeutic Options for a Rare Ovarian Cancer Subtype. J. Clin. Oncol..

[32] Letrozole with or without Paclitaxel and Carboplatin in Treating Patients with Stage II-IV Ovarian, Fallopian Tube, or Primary Peritoneal Cancer. NRG-GY019 Trial. https://clinicaltrials.gov/ct2/show/NCT04095364.

